# Supplementary material for: Selective therapeutic strategy for p53-deficient cancer by targeting dysregulation in DNA repair
Source: Commun Biol. 2021 Jul 12;4:862. doi: 10.1038/s42003-021-02370-0 (PMC8275734; doi:10.1038/s42003-021-02370-0)

TITLE: Selective therapeutic strategy for p53-deficient cancer by targeting dysregulation in DNA repair.

AUTHORS: Justin Zonneville, Moyi Wang, Mohammed M. Alruwaili, Brandon Smith, Megan Melnick, Kevin H. Eng, Thomas Melendy, Ben Ho Park, Renuka Iyer, Christos Fountzilas, and Andrei V. Bakin

pg. 1 TABLE of CONTENT

pg. 2 Supplementary Materials and Methods

pg. 3-4 Supplementary Tables 1-3

pg. 5-12 Supplementary Figures 1-8

pg. 13-21 Supplementary Figures, uncropped blots

Reagent or Resource	Source	Identifier	Dilution
Antibodies			
anti-GAPDH (rabbit, polyclonal)	Santa Cruz Biotechnology	Cat# sc-25778; RRID: AB_10167668	1:5000; IB
anti-Thymidylate Synthase (mouse monoclonal, Clone TS106)	Millipore	Cat# mab4130; RRID:AB_2210729	1:500; IB
anti-p53 (Mouse Monoclonal antibody, Clone DO-1)	Santa Cruz Biotechnology	Cat# sc-126; RRID:AB_628082	1:2000; IB
anti-p21 (CDKN1A) (C-19, rabbit polyclonal)	Santa Cruz Biotechnology	Cat# sc-397; RRID:AB_632126	1:1000; IB
anti-RAD51 (H-92, rabbit polyclonal)	Santa Cruz Biotechnology	Cat# sc-8349; RRID:AB_22253533	1:250; IF
anti-phospho-Ser15 p53 (rabbit, polyclonal)	Cell Signaling Technology	Cat# 9284; RRID:AB_331464	1:2000; IB
anti-PAR (mouse monoclonal anti-PADPr, Clone 10h)	Santa Cruz Biotechnology	Cat# sc-56198, RRID:AB_785249	1:250; IB
anti-PARP1 (Mouse monoclonal antibody, Clone 5a5)	Santa Cruz Biotechnology	Cat# sc-56197; RRID:AB_630080	1:500; IB
anti-phospho-Ser139 H2AX (γH2AX; rabbit polyclonal)	Abcam	Cat# ab-11174; RRID:AB_297813	1:2000; IB
anti-phospho-Ser139 H2AX (γH2AX; rabbit polyclonal)	Abcam	Cat# ab-11174; RRID:AB_297813	1:6000; IHC
anti-phospho-Ser139 H2AX (γH2AX; mouse mAb, JBW301)	Millipore	Cat# 05-636; RRID:AB_309864	1:1000; IF
anti-MDM2 (mouse monoclonal, Clone SMP14)	Santa Cruz Biotechnology	Cat# sc-965; RRID:AB_627920	1:500; IB
Goat Anti-Mouse IgG (H L)-HRP Conjugate antibody	Bio-Rad	Cat# 170-6516; RRID:AB_11125547	1:2000; IB
Goat Anti-Rabbit IgG (H L)-HRP Conjugate antibody	Bio-Rad	Cat# 170-6515; RRID:AB_11125142	1:2000; IB
anti-BrdU (Bu20a)	Dako	Cat# M0744; RRID:AB_10013660	1:100; IHC
Cleaved Caspase-3 (Asp175) Antibody	Cell Signaling Technology	Cat# 9661; RRID:AB_2341188	1:400; IHC
Rabbit monoclonal anti-Ki67 (clone SP6)	ThermoFisher	Cat#RM-9106-S1, RRID:AB_149792	1:100; IHC
Biotinylated secondary anti-rat antibody (goat)	BD Biosciences	Cat#559286, RRID:AB_397214	1:2000; IB

Reagents, Chemicals, siRNA and Cytokines		
Olaparib (Synonyms: AZD2281; KU0059436)	MedChemExpress	Cat# HY-10162
Talazoparib (Synonyms: BMN-673; LT-673)	MedChemExpress	Cat# HY-16106
TAS102 (Trifluridine/tipiracil hydrochloride mix)	MedChemExpress	Cat# HY-16478
Floxuridine (5-fluoro-2'-deoxyuridine; FdUrd)	MedChemExpress	Cat# HY-B0097
5-Fluorouracil	MedChemExpress	Cat# HY-90006
5-Ethynyl-2'-deoxyuridine (EdUrd)	Lumiprobe	Cat# 20540
5-Bromo-2'-deoxyuridine (BrdUrd)	MedChemExpress	Cat# HY-15910
CellTrace Violet Dye	Invitrogen	Cat#C34557
Hydroxyurea	Sigma	Cat#55291
TRIzol Reagent	Invitrogen	Cat# 15596-026
Cy3-azide	Lumiprobe	Cat#B1030
AFDye 488 Azide	Click Chemistry Tools, Scottsdale	Cat#1275-1
D-luciferin	Gold Biotechnology, St Louis	LUCK-1G
(2-Hydroxypropyl)-β-cyclodextrin (HPCD; Cavasol)	Sigma	Cat# 778907
Propidium iodide	Sigma	Cat#P4170
Hoechst 33342	Sigma	Cat#B2261
Dialyzed FBS	Gibco	Cat# A33820
Scramble siRNA	Sigma	Cat# SIC003-10nmol
siRNA-p53 GAGGUUGGCUCUGACUGUAdTdT	Sigma	Cat#SASI_Hs02_00302766/TP53
Protease Inhibitor Cocktail	Roche	Cat# 11836153001
Nitrocellulose membranes	Bio-Rad	Cat# 162-0112
ECL chemiluminescent reagent	Pierce	Cat# 32209

Supplementary Table 1. IC50 and Combination Index for 5-fluorodeoxyuridine (5FdUrd).

Cell Line	TP53 status	FdUrd, IC50 (μM)	FdUrd+Ola, IC50 (μM)	Ola, IC50 (μM)	CI-index
MCF10A	WT	2.32 ± 1.31	2.74 ± 0.39	23.84 ± 4.03	1.20
WI-38	WT	3.76 ± 0.39	23.26 ± 2.46	4.26 ± 3.84	6.30
EMT6	WT	0.07 ± 0.02	0.09 ± 0.02	5.66 ± 0.32	1.37
CAL51	WT	0.11 ± 0.01	0.11 ± 0.01	8.96 ± 0.02	1.06
MDA-MB-231	Mut	4.61 ± 0.88	0.51 ± 0.24	17.30 ± 5.07	0.14
MDA-MB-468	Mut	1.72 ± 0.68	0.14 ± 0.08	6.90 ± 1.01	0.15
4T1	Mut	0.62 ± 0.30	0.14 ± 0.05	7.70 ± 4.93	0.29

Combination assays included 0.5μM Olaparib.

Supplementary Table 2. IC50 and Combination Index for 5-fluorouracil (5FU).

Cell Line	TP53 status	FU, IC50 (μM)	FU+Ola, IC50 (μM)	Ola, IC50 (μM)	CI-index
MCF10A	WT	25.03 ± 0.40	23.82 ± 0.25	23.84 ± 4.03	0.97
WI-38	WT	5.11 ± 0.70	5.80 ± 0.86	4.26 ± 3.84	1.25
EMT6	WT	0.89 ± 0.22	1.40 ± 0.64	5.66 ± 0.32	1.66
MDA-MB-231	Mut	11.33 ± 1.82	12.28 ± 3.53	17.30 ± 5.07	1.12
MDA-MB-468	Mut	18.02 ± 2.62	20.57 ± 2.07	6.90 ± 1.01	1.21
4T1	Mut	0.56 ± 0.06	0.55 ± 0.12	7.70 ± 4.93	1.05

Combination assays included 0.5μM Olaparib.

Supplementary Table 3. IC50 and Combination Index for TAS102.

Cell Line	TP53 status	TAS102, IC50, µM	TAS+Ola, IC50 (µM)	Ola, IC50 (µM)	CI-index
MCF10A	WT	3.30 ± 0.51	3.11 ± 0.51	23.84 ± 4.03	0.99
WI-38	WT	2.08 ± 0.65	4.94 ± 0.81	4.26 ± 3.84	2.49
EMT6	WT	0.33 ± 0.07	0.28 ± 0.06	5.66 ± 0.32	0.94
MDA-MB-231	Mut	2.11 ± 0.18	0.54 ± 0.07	17.30 ± 5.07	0.28
MDA-MB-468	Mut	5.68 ± 0.82	0.08 ± 0.02	6.90 ± 1.01	0.09
4T1	Mut	0.31 ± 0.01	0.10 ± 0.01	7.70 ± 4.93	0.39

Combination assays included 0.5µM Olaparib.

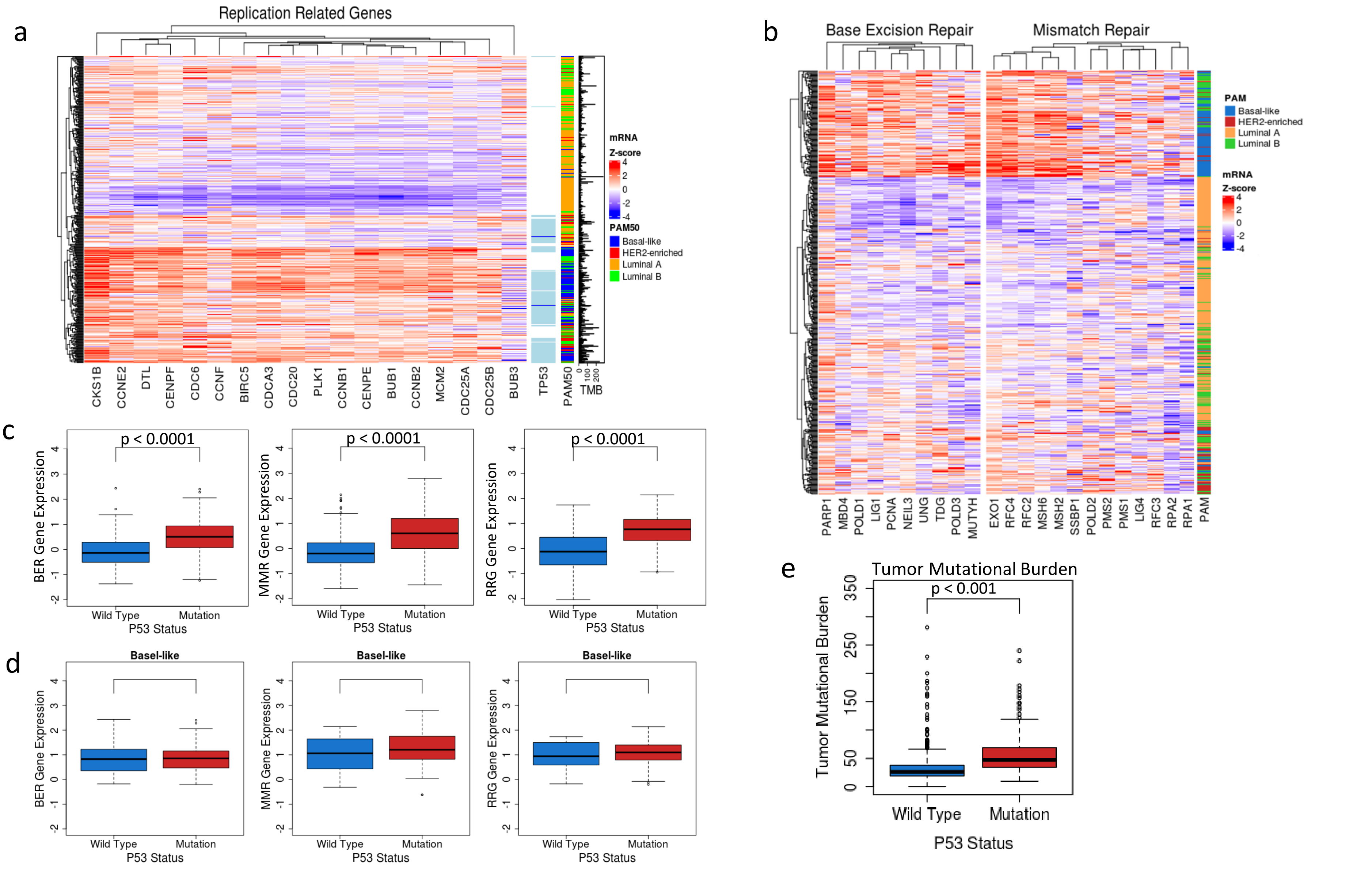

Supplementary Fig.1. Genomics of Replication-Related Genes (RRG) and DNA repair (BER/MMR) genes in breast cancer. (**a**) Expression profiles of replication-related genes (S-phase and M-phase) in Breast Cancer subtypes (TCGA BC dataset). Gene lists are designed using Cyclebase_3.0 database. (**b**) Clustering analysis of BER and MMR Gene lists are derived from KEGG. (**c-d**) Average expression of BER, MMR, and RRG genes and *TP53* status in breast cancer (**c**) or in TNBC/Basal-like group (**d**) (TCGA BC dataset). Plots show sample means ± 1sd. (**e**) Tumor mutational burden in Breast Cancer (TCGA BC dataset).

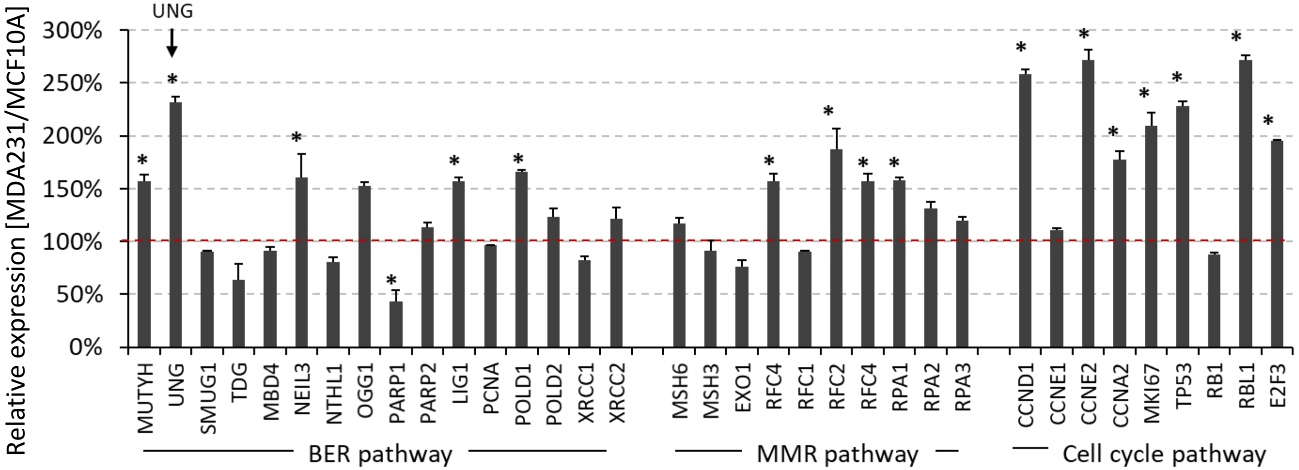

Supplementary Fig. 2. Comparison of gene expression for BER, MMR, and replication-related pathway genes in p53-mutant MDA-MB-231 cells relative to p53 wild-type MCF10A cells.

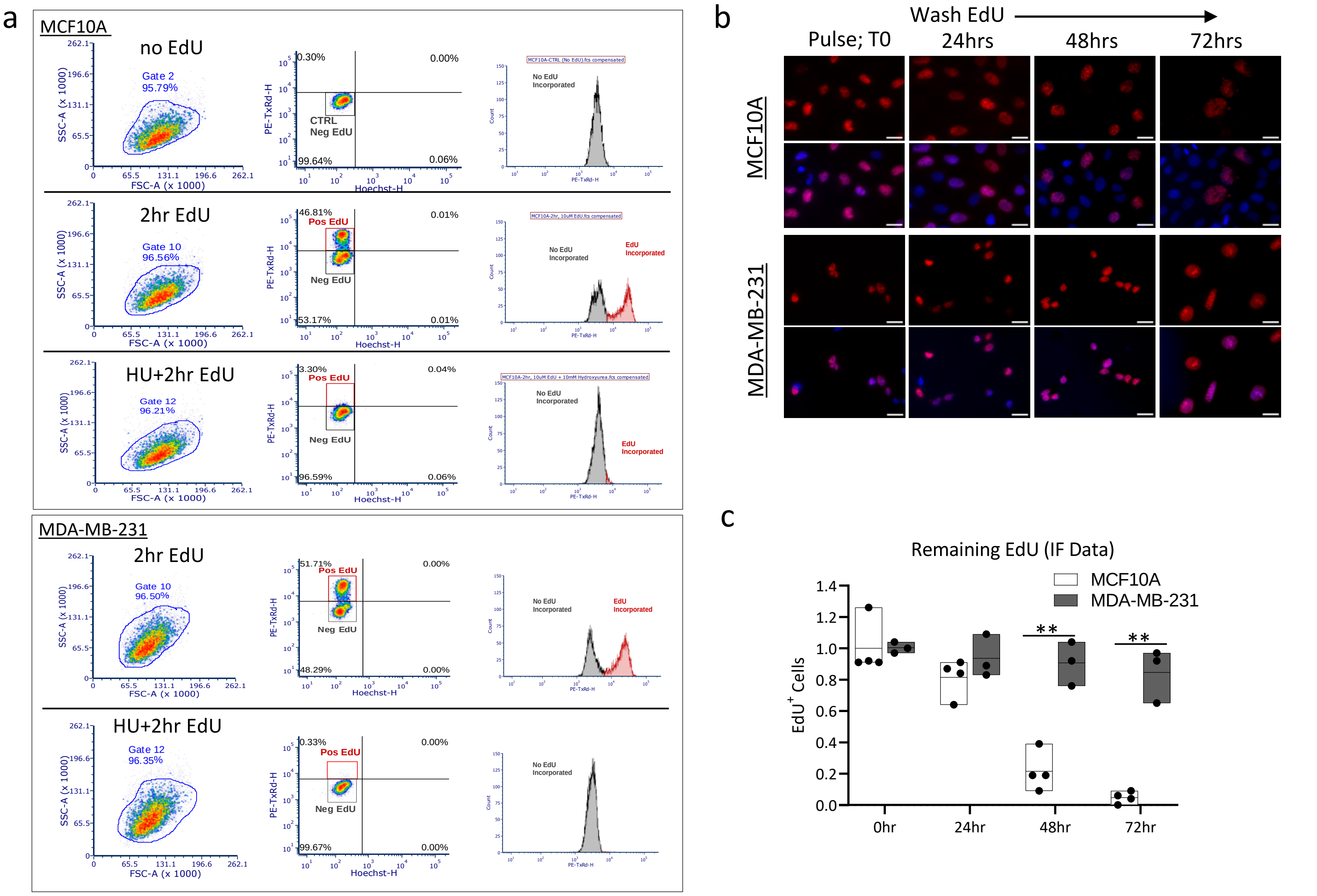

Supplementary Fig. 3. Assessment of ethynyl-deoxyuracil (EdU) incorporation in cell lines. (**a**) Flow Cytometry of p53wt MCF10A and p53mt MDA-MB-231 cells which were pulse-labeled with 10μM 5-ethynyl-2'-deoxy-uridine (EdUrd) for 2 hrs -/+ hydroxyurea (HU). (**b**) Fluorescence microscopy images: cells were pulse-labeled with 10μM EdUrd for 2 hrs followed by wash and incubation for indicated time. Cells were fixed and stained for EdU using click-it chemistry. Images were taken with 60X lens, bar=20μm. (**c**) Quantification of EdU-positive cells from (**b**) relative to a total cell number. Comparison was made using the Log rank test (**, P<0.01).

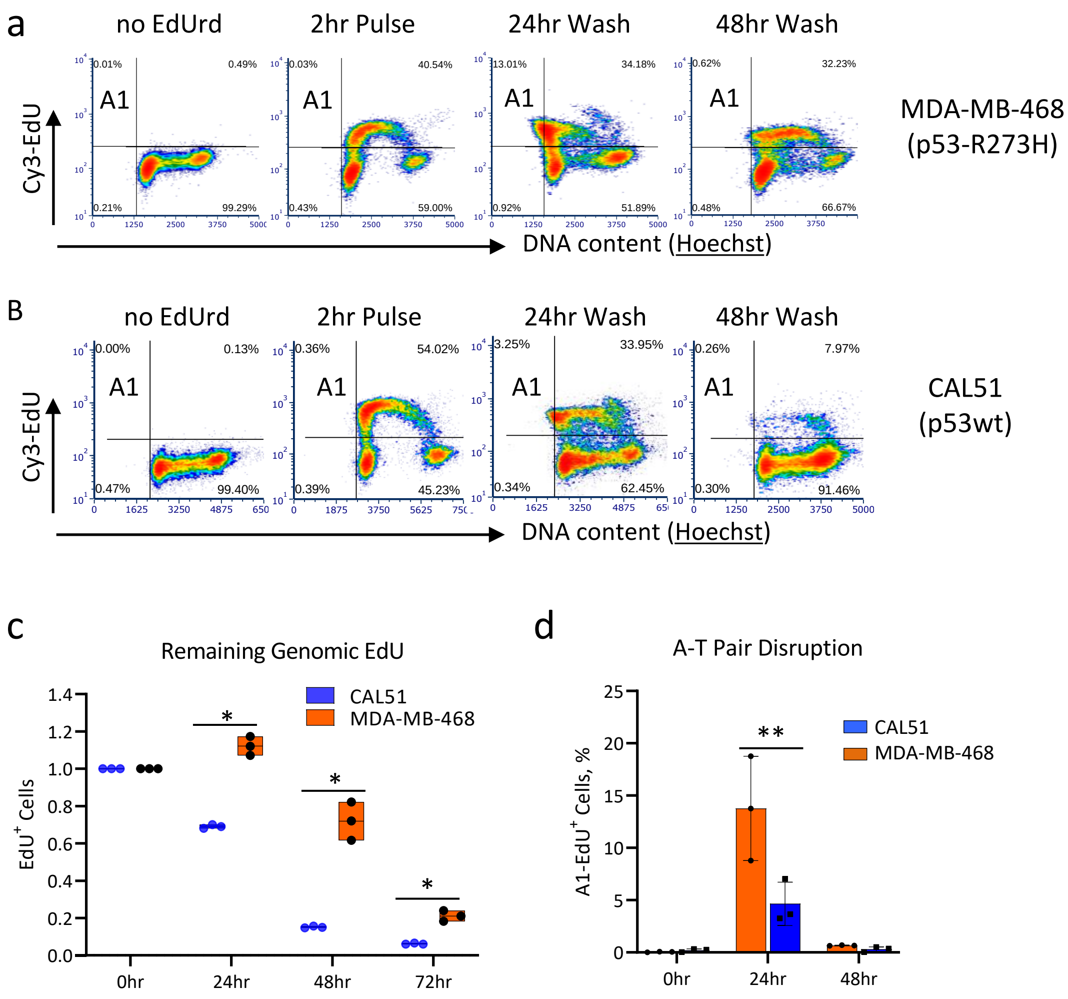

Supplementary Fig. 4. Removal of genomic EdU in MDA-MB-468 (p53mt) and CAL51 (p53wt) cell lines. Cells were incubated with 10µM EdUrd for 2 hours, washed, and incubated in EdUrd-free media for indicated time. (**a-b**) Cells were stained for EdU and DNA content by using Cy3-azide and Hoechst-33342. (**c**) Quantification of remaining genomic EdU in MDA-MB-468 and CAL51 cells after EdUrd-pulse labeling. (**d**) EdU^{+} cells with reduced Hoechst fluorescence (upper-left quartile A1). Comparison was made using the Log rank test (**, P<0.01; *, P<0.05).

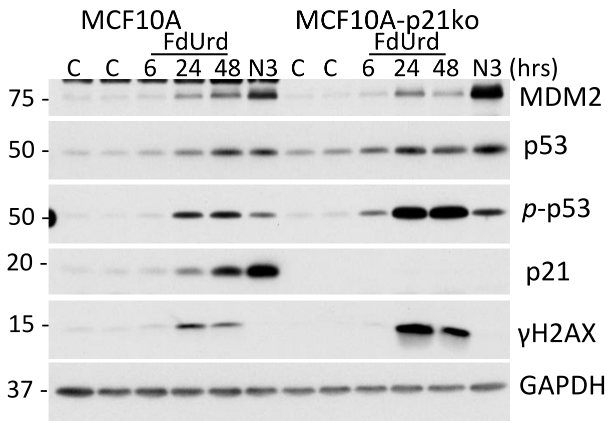

Supplementary Fig. 5. Immunoblots of whole-cell extracts from p21 wild-type and p21-knockout MCF10A cell lines. MCF10A cells carrying deletion of p21 (p21ko) and control p21wt MCF10A cells were treated with 5µM FdUrd or 3µM Nutlin-3A for 6, 24, or 48 hrs. Immunoblot analysis for DNA damage marker γH2AX, phospho-Ser15 p53, p21 and MDM2.

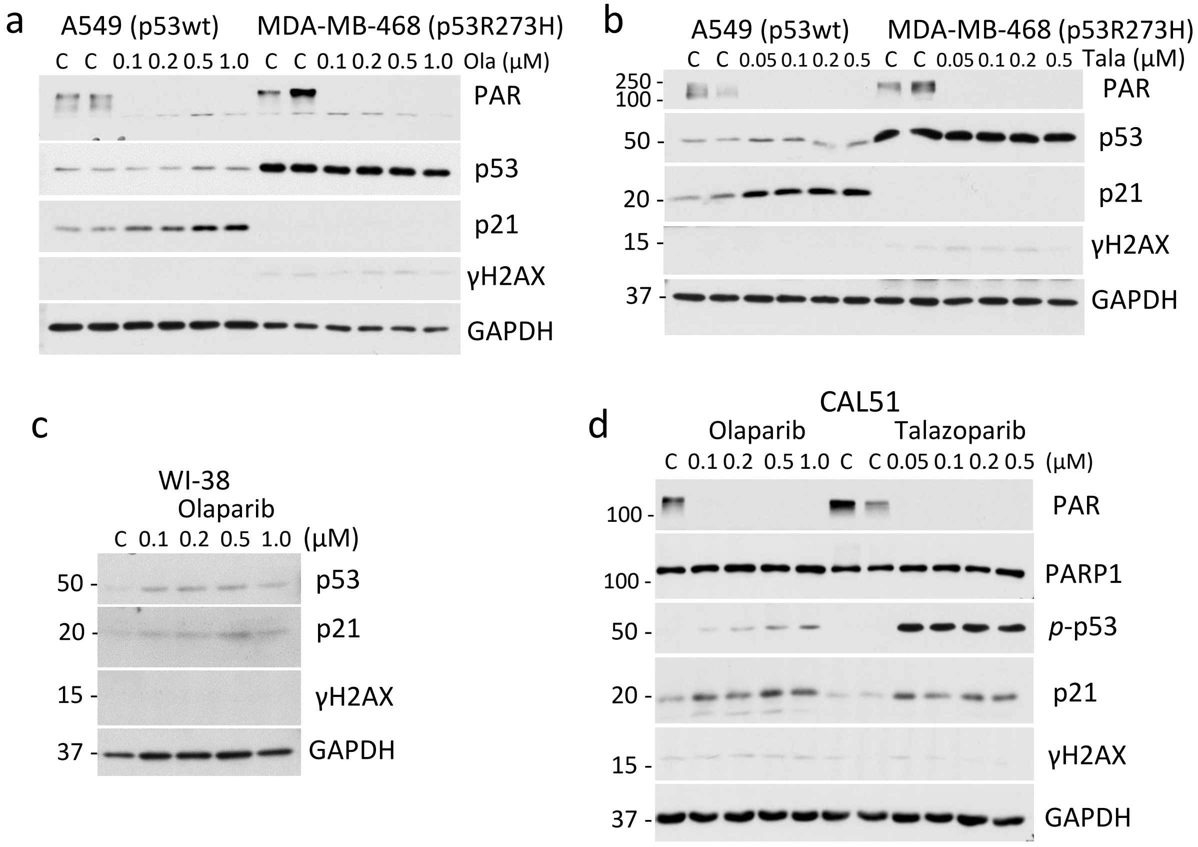

Supplementary Fig. 6. Immunoblots of whole-cell extracts from p53 wild-type A549, WI-38 and CAL51 cell lines and p53mt breast cancer MDA-MB-468 cell line. (**a-d**) cells were treated with olaparib (100-1000nM) and talazoparib (50-500nM) for 24 hours. (**d**) Human TNBC p53wt CAL51 cell line was treated with olaparib or talazoparib as described in (**a**).

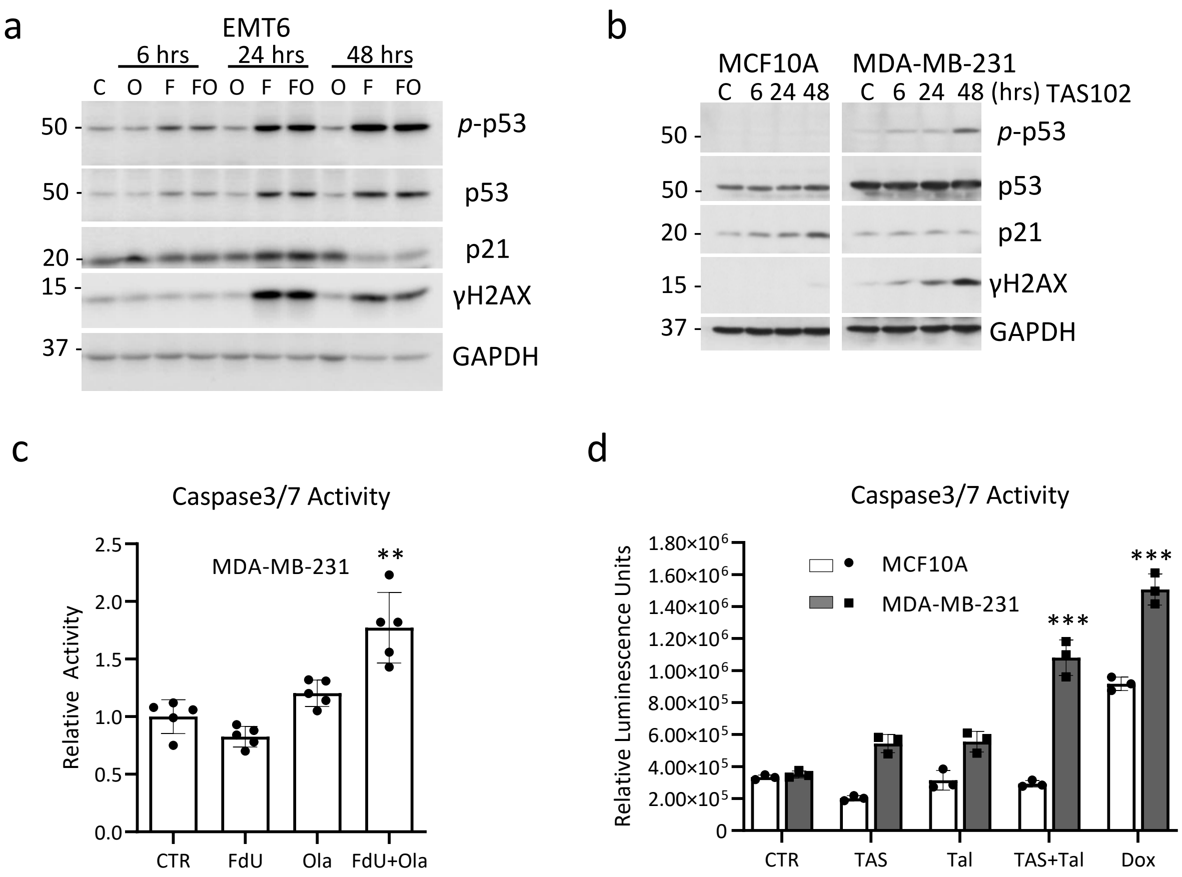

Supplementary Fig. 7. Responses to the drug combination in p53 wild-type and mutant cell lines. (**a**) Mouse mammary carcinoma p53wt EMT6 cells were treated with floxuridine (F, 5μM), olaparib (O, 0.5μM) or their combination (FO) for indicated time. (**b**) Cells were treated with 2μM TAS102 for indicated time. (**c**) Relative Caspase 3/7 activity in MDA-MB-231 treated with vehicle-control, 2μM FdUrd (FdU), 300nM olaparib (Ola), and the drug combination for 48 hours. (**d**) Relative Caspase 3/7 activity (RLU) in MDA-MB-231 and MCF10A cells treated with vehicle-control, 2μM TAS102 (TAS), 100nM talazoparib (Tal), and the drug combination for 48 hours. Cells were treated with 0.2μM doxorubicin for 24 hours, where is indicated. Comparison was made using the Log rank test (***, P<0.001; **, P<0.01).

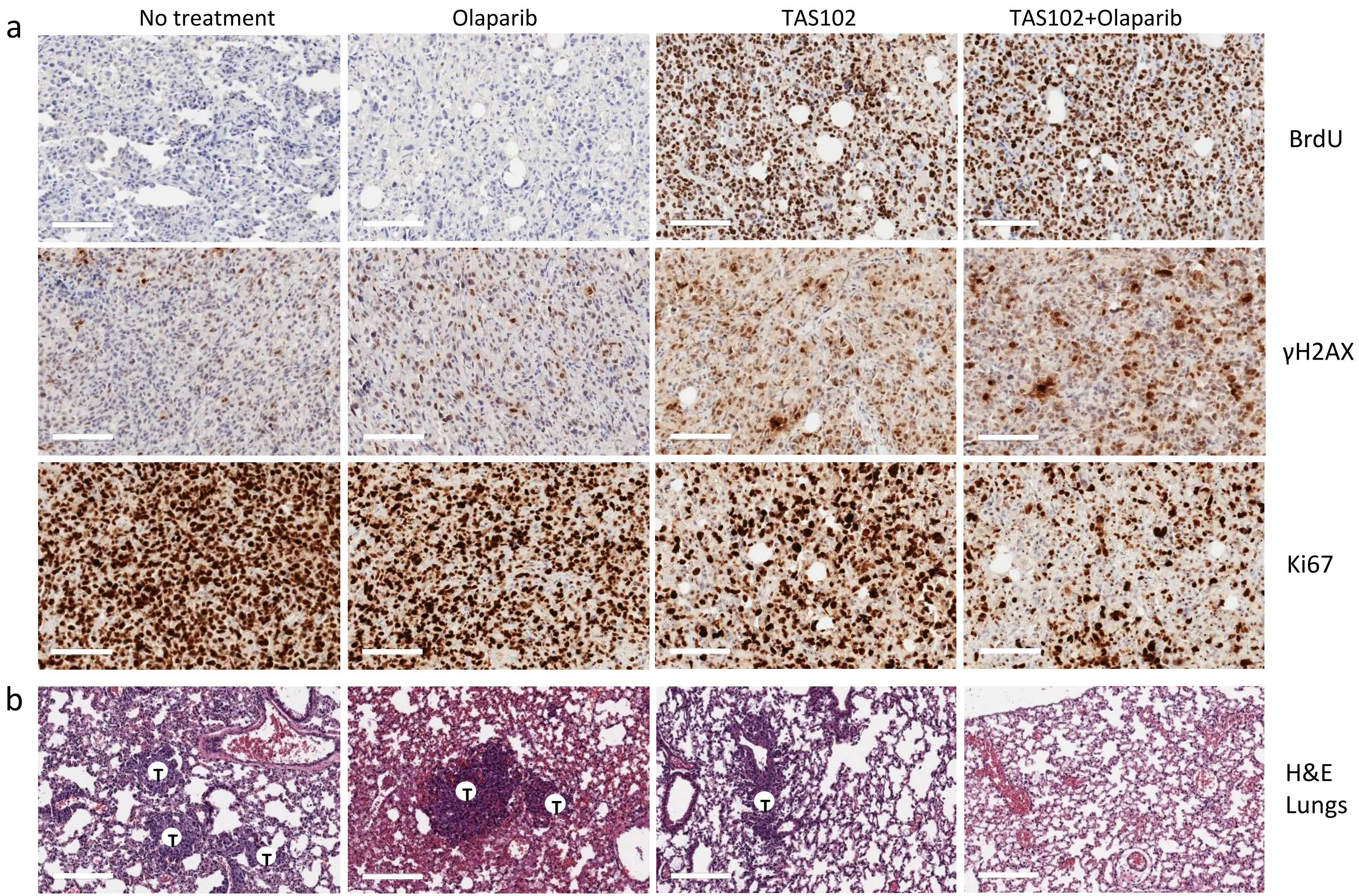

Supplementary Fig. 8. Histology of TNBC tumor and mouse lungs in tumor-bearing mice. (**a**) Female SCID mice inoculated with breast cancer p53mt MDA-MB-231 tumors in a mammary fat pad were treated with TAS102, olaparib, or their combination. Incorporation of trifluorothymidine, TFT, (a component of TAS102) was detected using anti-BrdU antibodies, DNA damage response with antibodies to γH2AX, and proliferation with antibodies to Ki67. Images were taken at 200x magnification, scale bar 100µm. (**b**) Lung sections were stained by H&E in tumor-bearing mice treated as indicated. Images were taken at 100x, scale bar 200µm.

Figure 2F

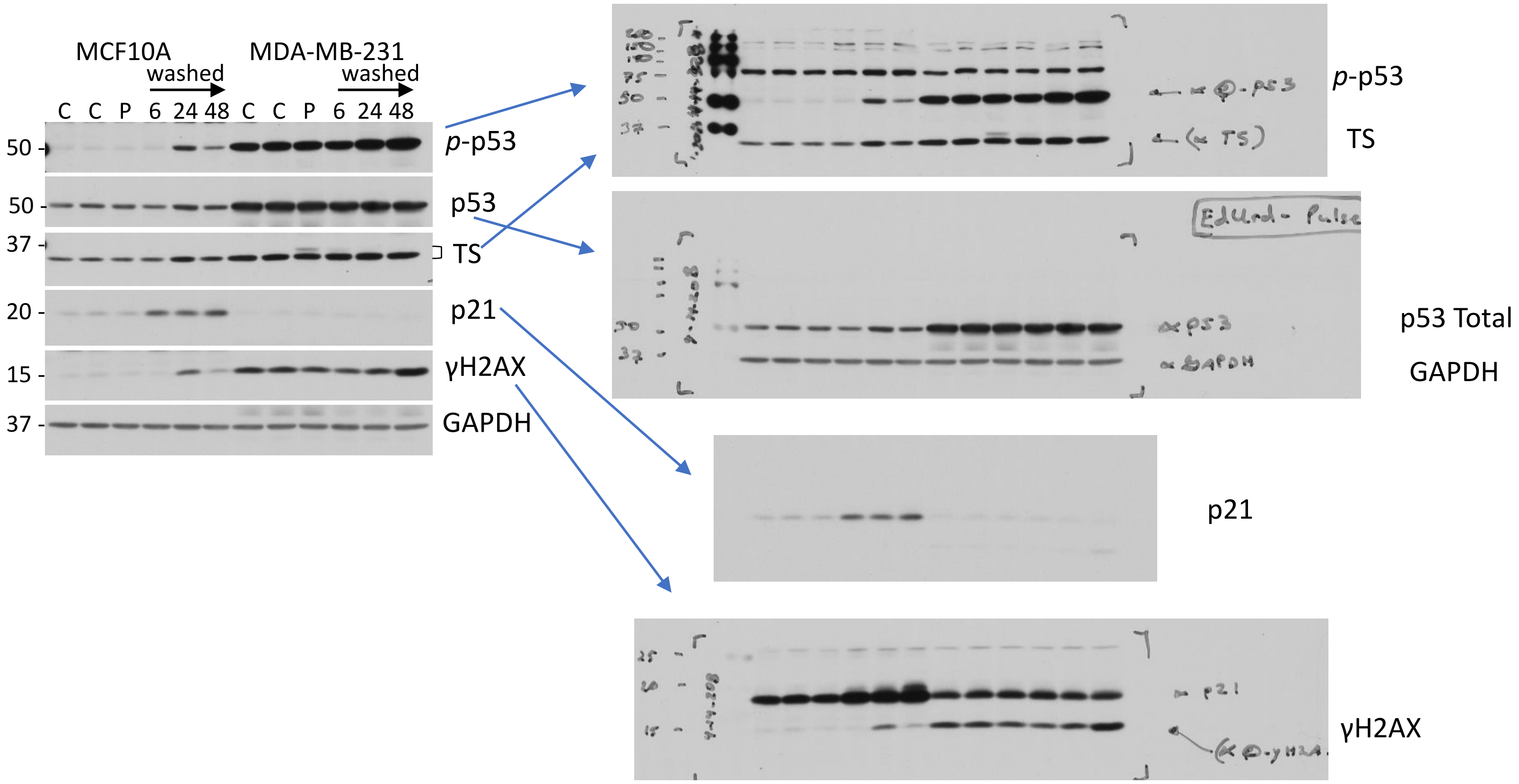

MCF10A
MDA-MB-231
washed
washed
C C P 6 24 48 C C P 6 24 48
50
50
37
20
15
37
p-p53
p53
TS
p21
γH2AX
GAPDH
p-p53
TS
p53 Total
GAPDH
p21
γH2AX

Figure 3A

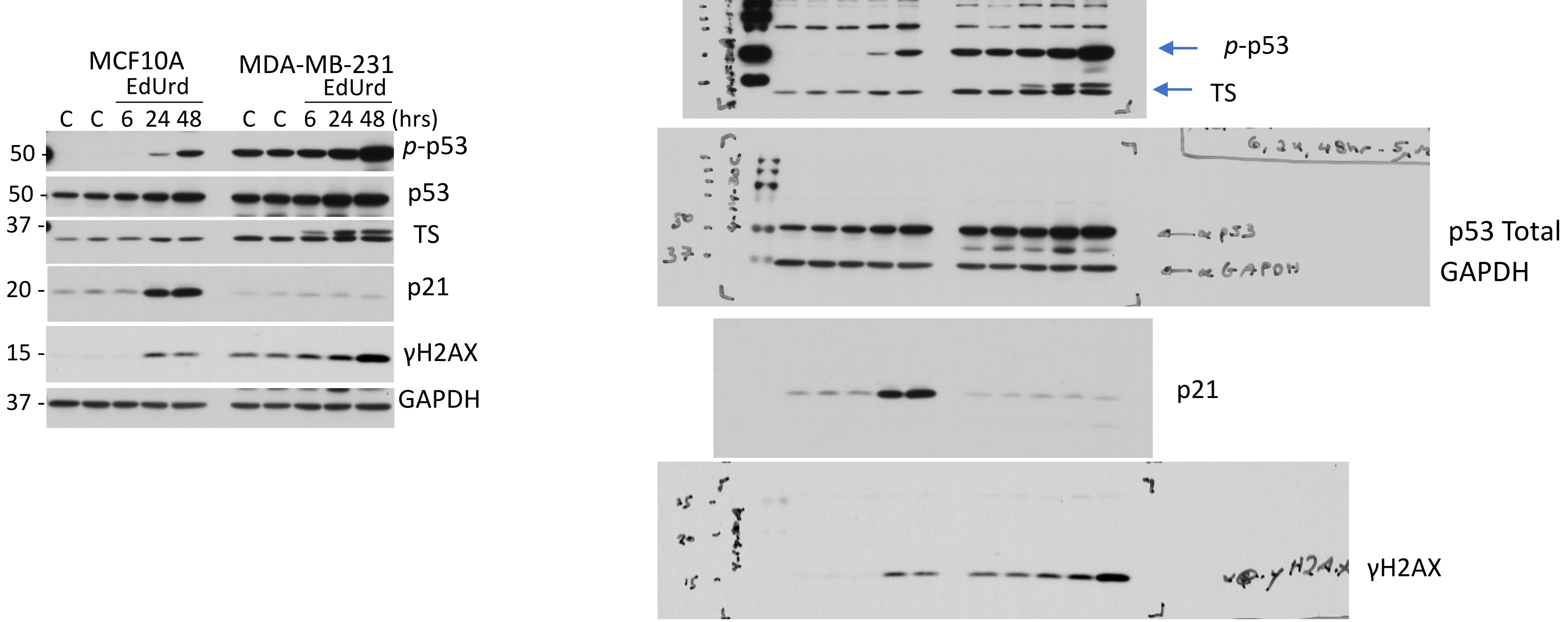

Figure 3B

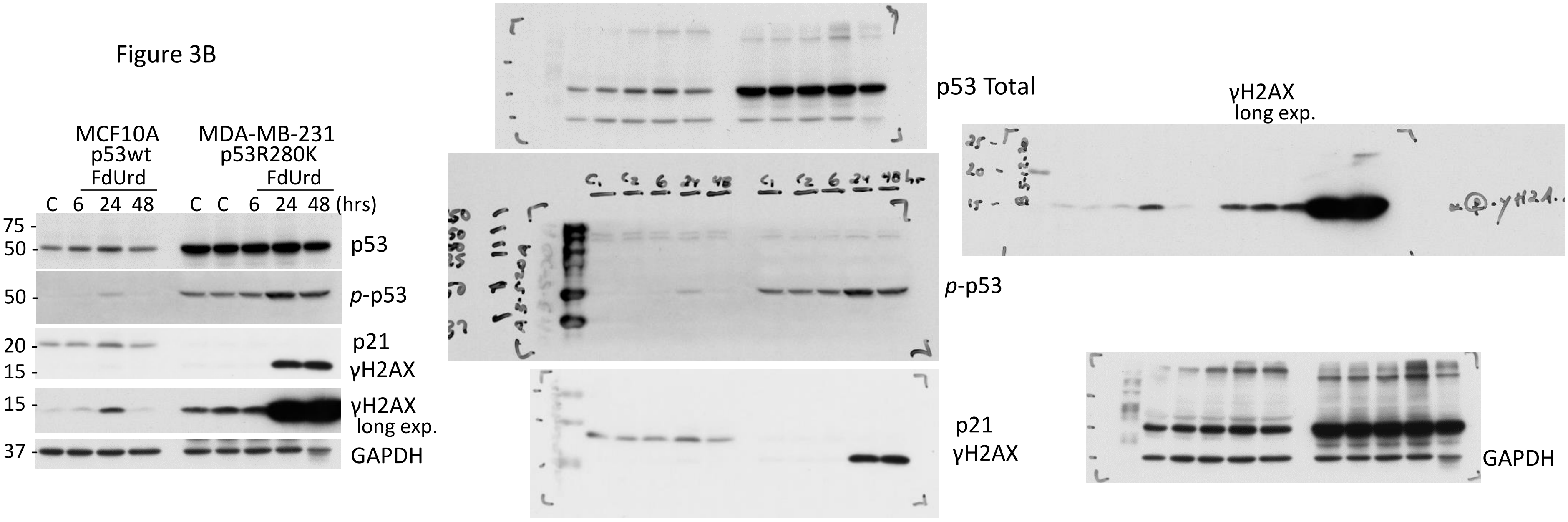

Figure 3C

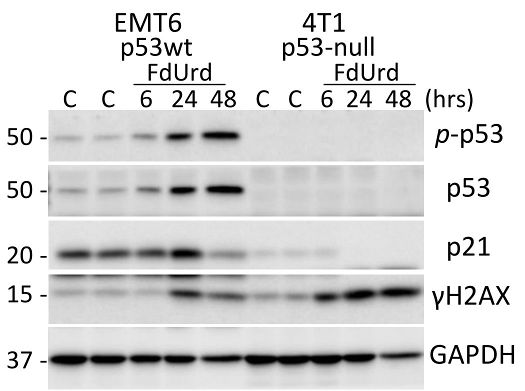

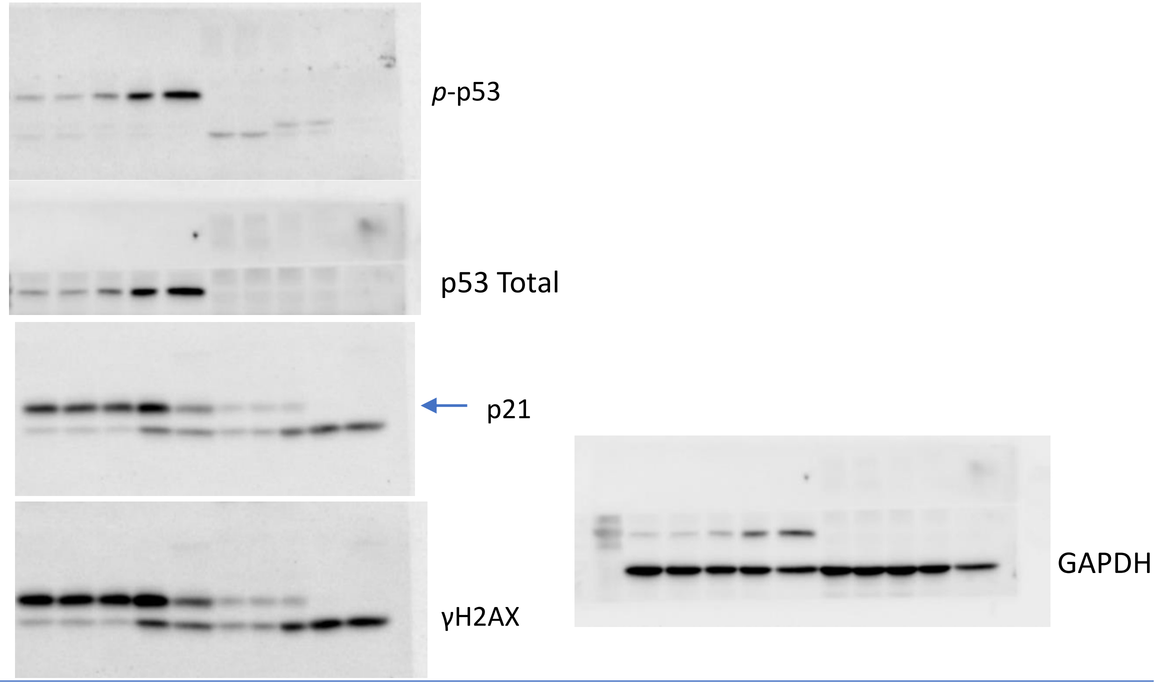

Figure 3D

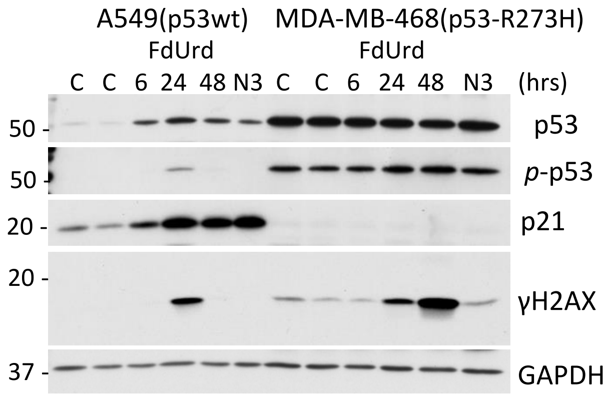

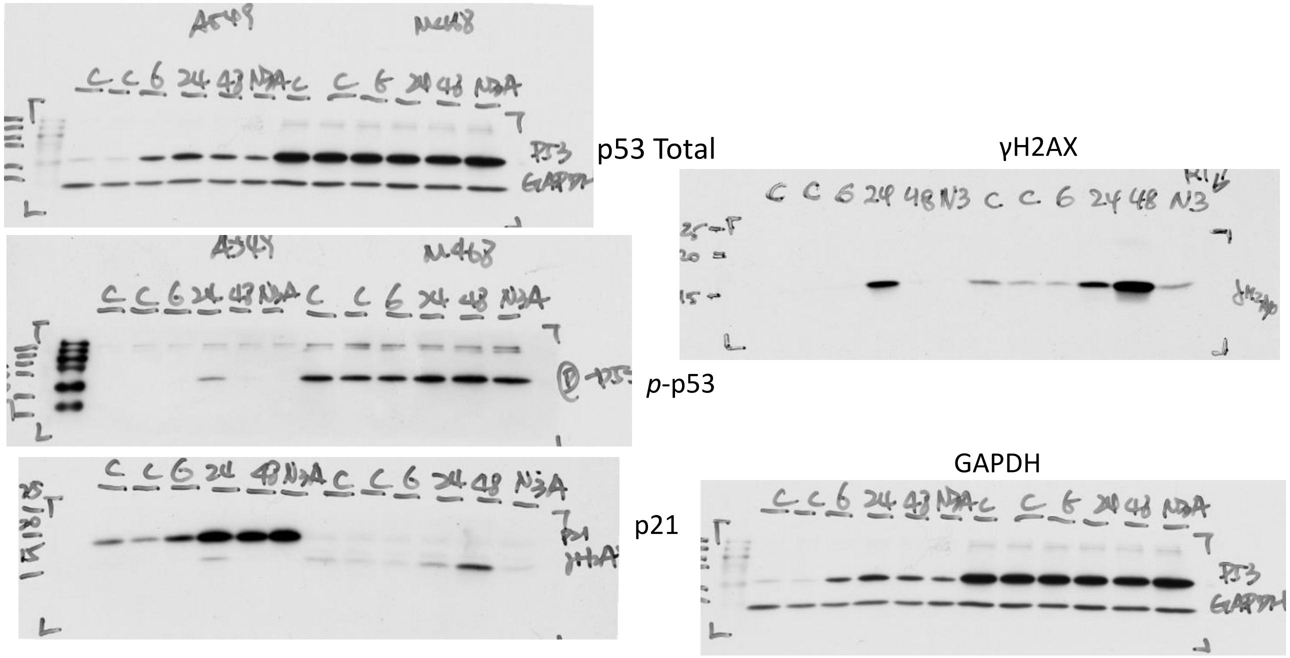

Figure 3E

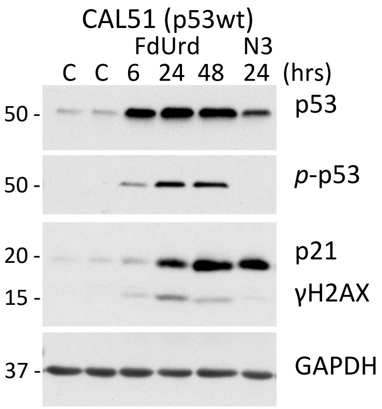

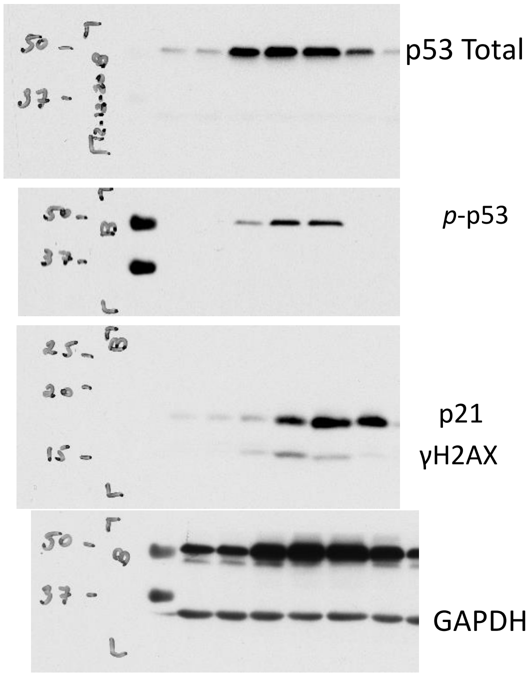

Figure 3F

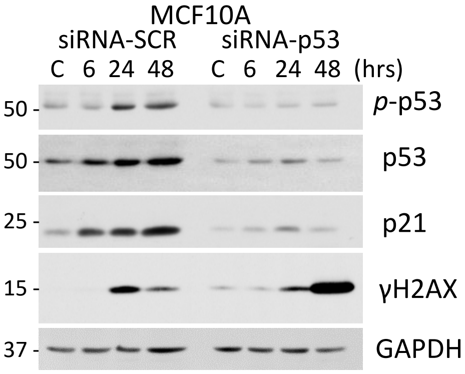

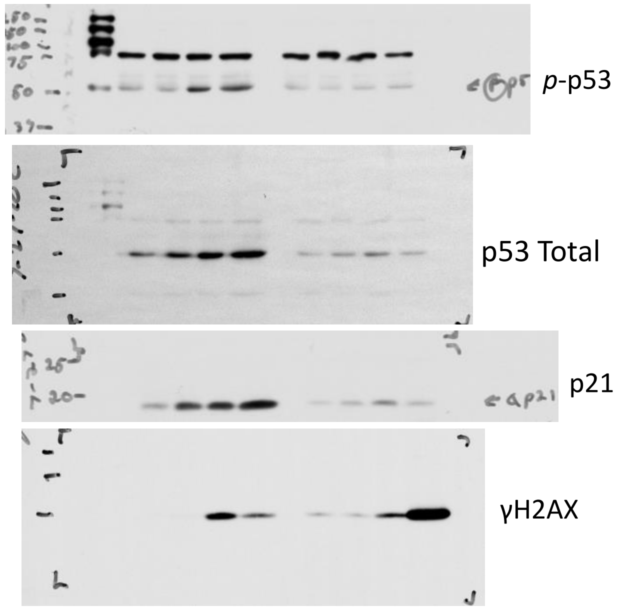

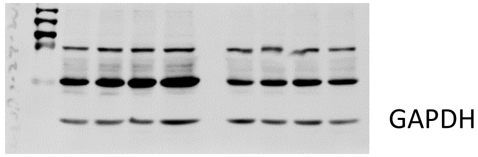

Figure 4B

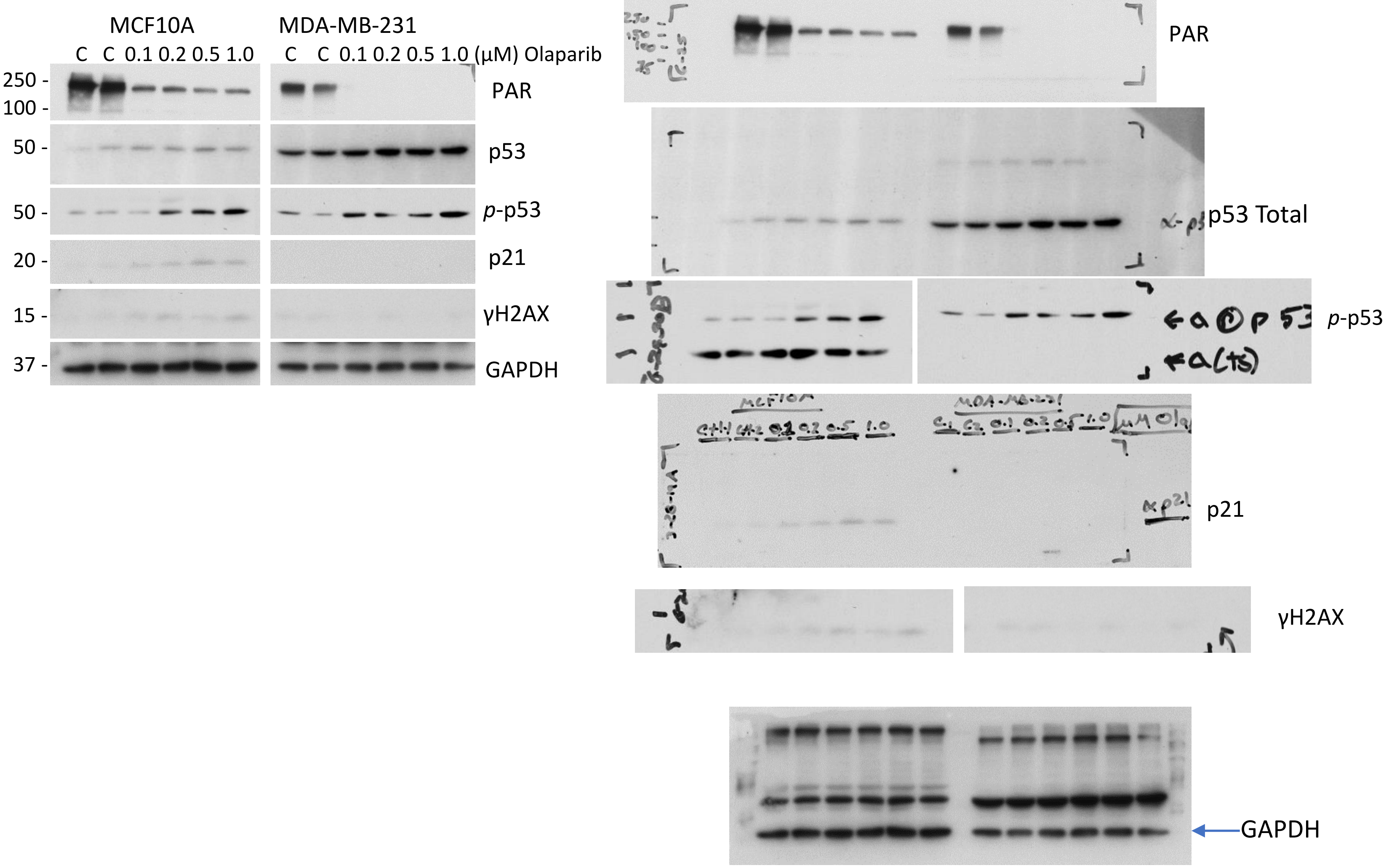

MCF10A
MDA-MB-231
C C 0.1 0.2 0.5 1.0 C C 0.1 0.2 0.5 1.0 (μM) Olaparib
250 -
100 -
PAR
50 -
p53
50 -
p-p53
20 -
p21
15 -
γH2AX
37 -
GAPDH
PAR
p53 Total
p-p53
p21
γH2AX
GAPDH

Figure 4C

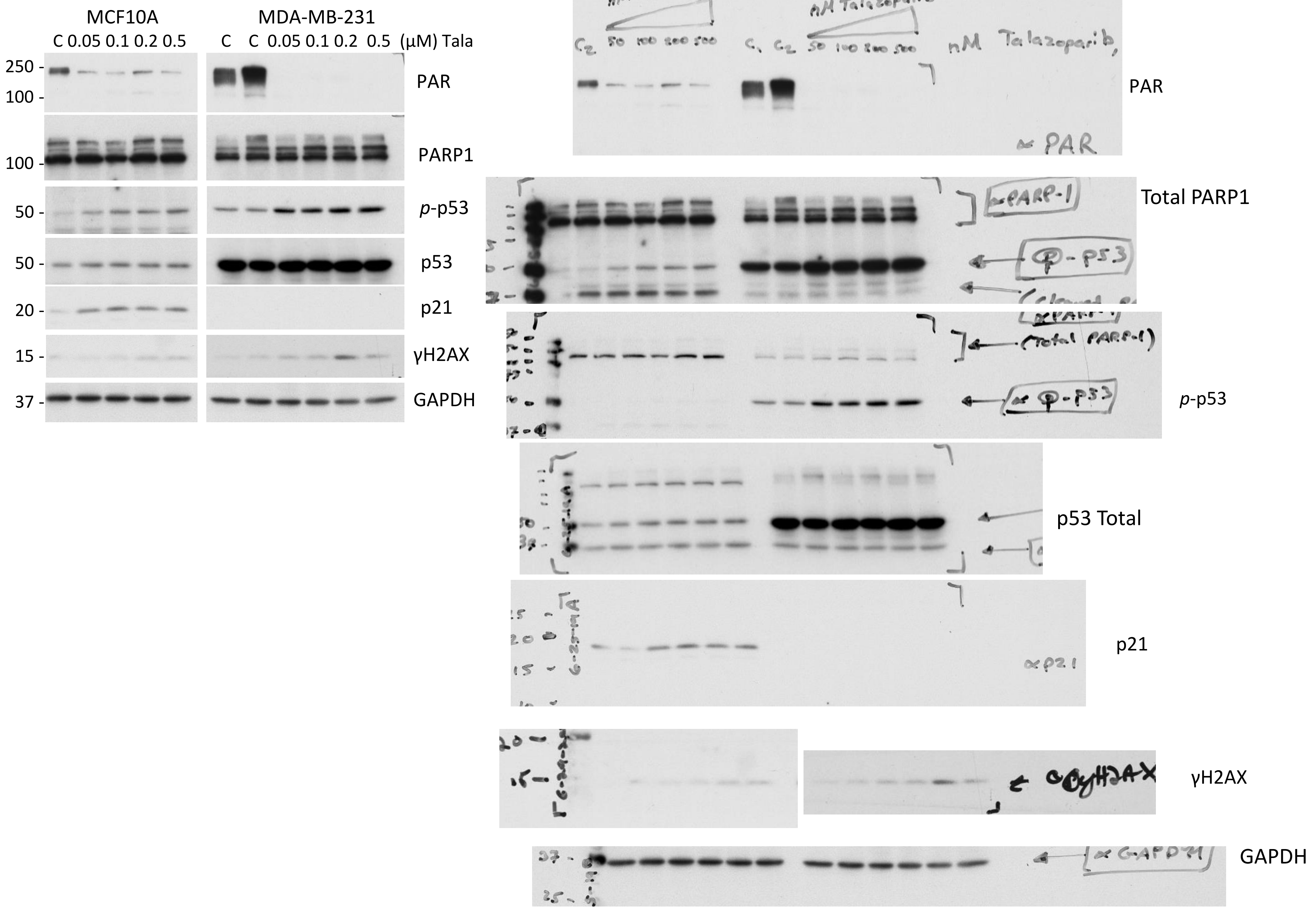
MCF10A
MDA-MB-231
C 0.05 0.1 0.2 0.5
C C 0.05 0.1 0.2 0.5 (μM) Tala
250
100
100
50
50
20
15
37
PAR
PARP1
p-p53
p53
p21
γH2AX
GAPDH
PAR
Total PARP1
p-p53
p53 Total
p21
γH2AX
GAPDH

Figure 5A

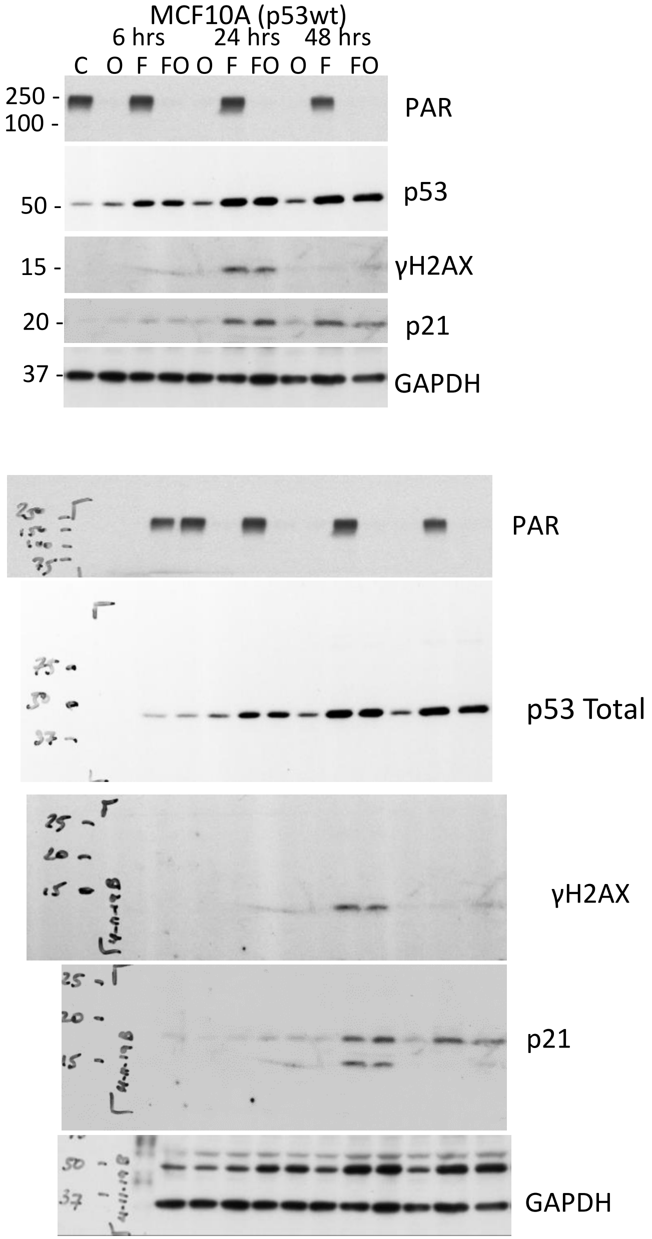

Figure 5B

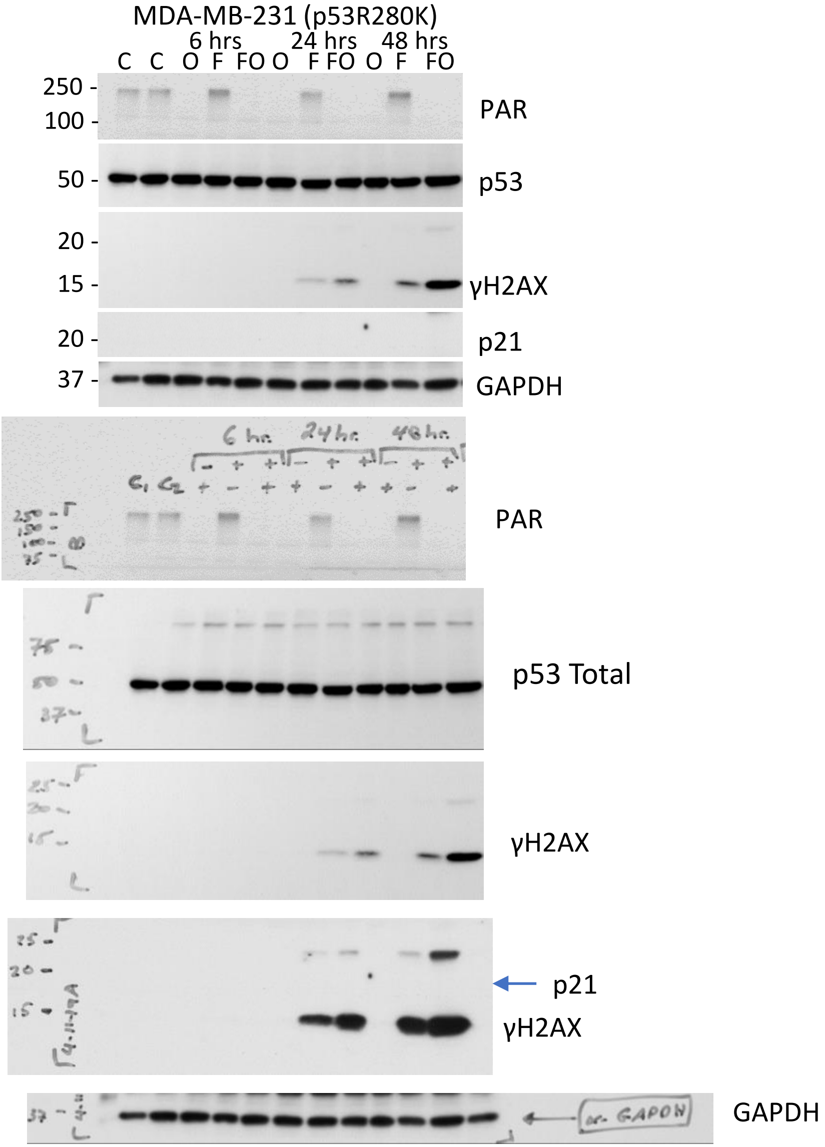

Figure 5C

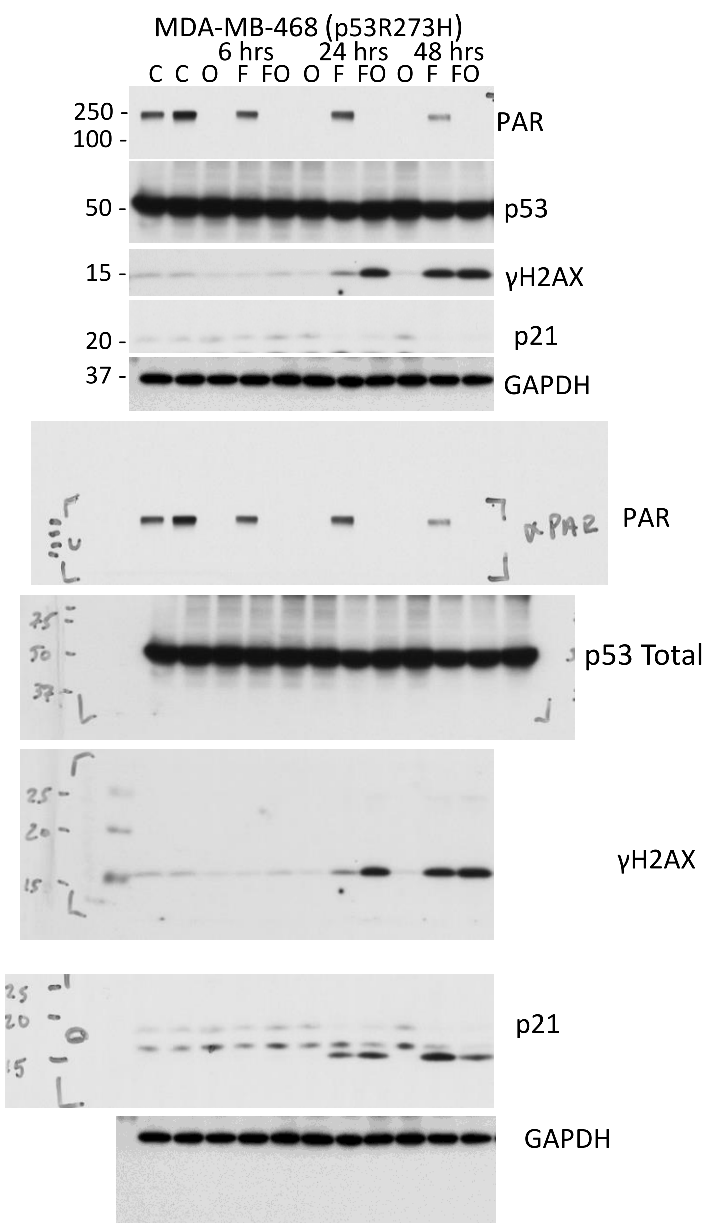

Figure 5D

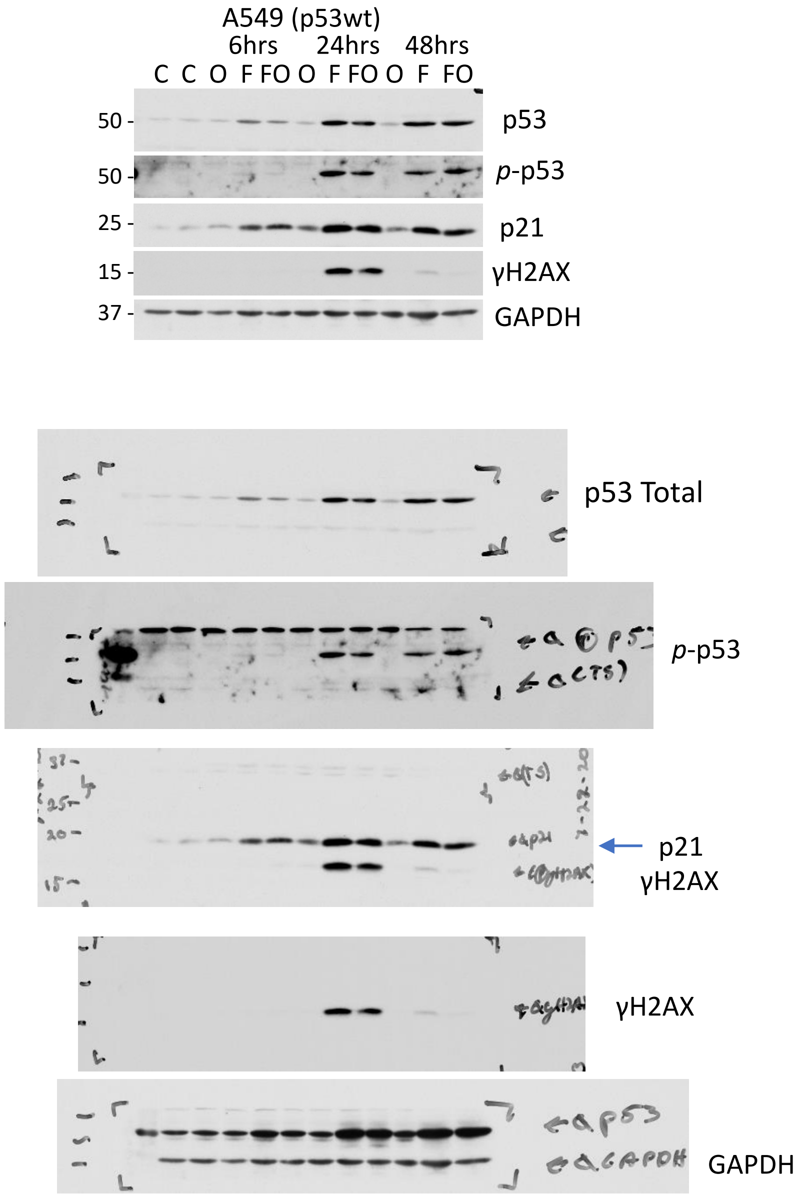

Figure 5E

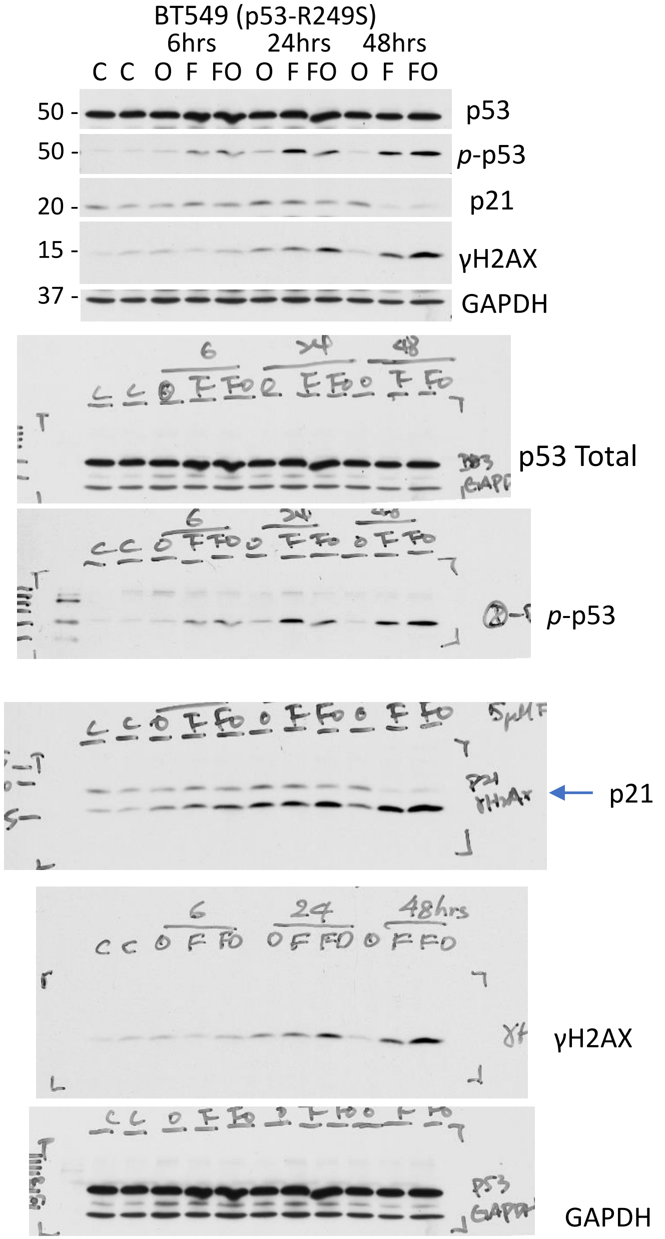

Figure 5F

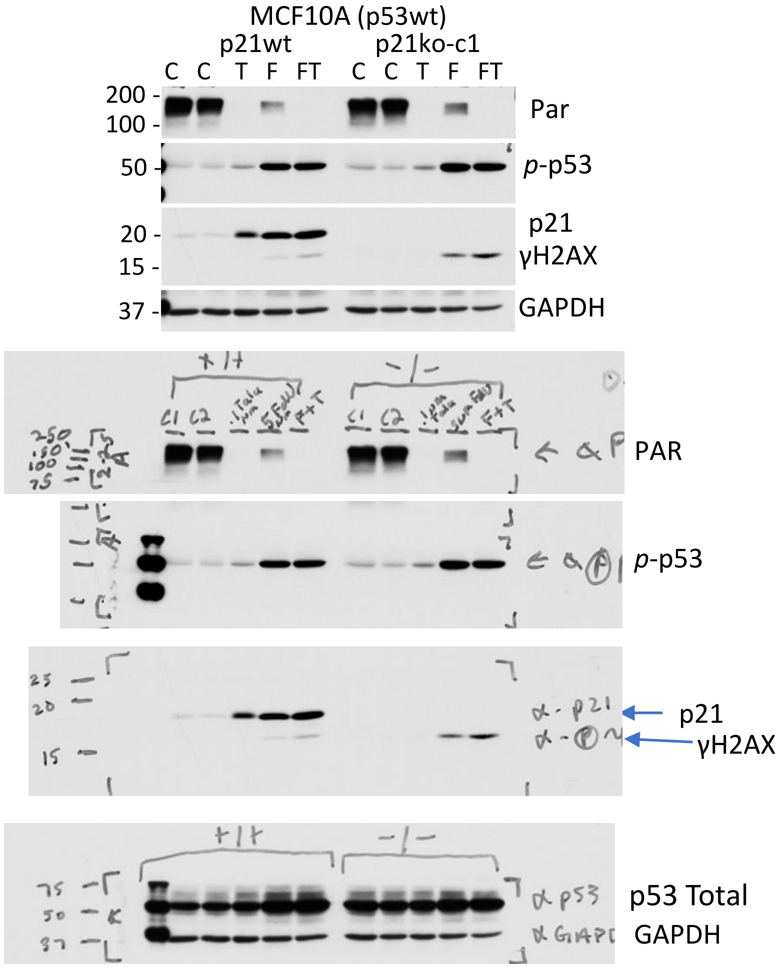

Supplement: Supplementary file 2 — Supplementary Information [file 42003_2021_2370_MOESM2_ESM.pdf]
