# Supplementary material for: Selective therapeutic strategy for p53-deficient cancer by targeting dysregulation in DNA repair
Source: Commun Biol. 2021 Jul 12;4:862. doi: 10.1038/s42003-021-02370-0 (PMC8275734; doi:10.1038/s42003-021-02370-0)
Supplement: Supplementary file 3 — Description of Additional Supplementary Files [file 42003_2021_2370_MOESM3_ESM.pdf]

**Description of Additional Supplementary Files**

**File name:** Supplementary Data 1
**Description:** Datasets for Figure 1 panels.

**File name:** Supplementary Data 2
**Description:** Data source for Figures in the main text.
