# Supplementary material for: Selective therapeutic strategy for p53-deficient cancer by targeting dysregulation in DNA repair
Source: Commun Biol. 2021 Jul 12;4:862. doi: 10.1038/s42003-021-02370-0 (PMC8275734; doi:10.1038/s42003-021-02370-0)
Supplement: Supplementary file 6 — Reporting Summary [file 42003_2021_2370_MOESM6_ESM.pdf]

Corresponding author(s): DBPR; COMMSBIO-20-2529A

Last updated by author(s): May 28, 2021

Reporting Summary

Nature Research wishes to improve the reproducibility of the work that we publish. This form provides structure for consistency and transparency in reporting. For further information on Nature Research policies, see our Editorial Policies and the Editorial Policy Checklist.

Statistics

For all statistical analyses, confirm that the following items are present in the figure legend, table legend, main text, or Methods section.

n/a	Confirmed	
☐	☒	The exact sample size (n) for each experimental group/condition, given as a discrete number and unit of measurement
☒	☐	A statement on whether measurements were taken from distinct samples or whether the same sample was measured repeatedly
☐	☒	The statistical test(s) used AND whether they are one- or two-sided<br>*Only common tests should be described solely by name; describe more complex techniques in the Methods section.*
☒	☐	A description of all covariates tested
☐	☒	A description of any assumptions or corrections, such as tests of normality and adjustment for multiple comparisons
☒	☐	A full description of the statistical parameters including central tendency (e.g. means) or other basic estimates (e.g. regression coefficient) AND variation (e.g. standard deviation) or associated estimates of uncertainty (e.g. confidence intervals)
☐	☒	For null hypothesis testing, the test statistic (e.g. F, t, r) with confidence intervals, effect sizes, degrees of freedom and P value noted<br>*Give P values as exact values whenever suitable.*
☒	☐	For Bayesian analysis, information on the choice of priors and Markov chain Monte Carlo settings
☒	☐	For hierarchical and complex designs, identification of the appropriate level for tests and full reporting of outcomes
☒	☐	Estimates of effect sizes (e.g. Cohen's d, Pearson's r), indicating how they were calculated

Our web collection on statistics for biologists contains articles on many of the points above.

Software and code

Policy information about availability of computer code

Data collection	FACSDiva (Version 6.1.3); MetaVue imaging software (v7.7.3, Molecular Devices); VERITAS microplate luminometer with version 1.9.3 software; cBioportal.
Data analysis	FCS Express 7 (Version 7.04.0016); HeskaView Integrated Software version 2.5.2; ImageJ software version 1.52C; GraphPad Prism 8 (Version 8.4.2); ModFit Lt software (Version 5.0.9); Microsoft Excel (Office 365 MSO); R/Rstudio 4.0.3.

For manuscripts utilizing custom algorithms or software that are central to the research but not yet described in published literature, software must be made available to editors and reviewers. We strongly encourage code deposition in a community repository (e.g. GitHub). See the Nature Research guidelines for submitting code & software for further information.

Data

Policy information about availability of data

All manuscripts must include a data availability statement. This statement should provide the following information, where applicable:
- Accession codes, unique identifiers, or web links for publicly available datasets
- A list of figures that have associated raw data
- A description of any restrictions on data availability

The heat-map data that support the findings of this study are generated using the cBIoportal for Cancer Genomics at http://www.cbioportal.org/ and the breast cancer TCGA dataset (Project ID: TCGA-BRCA; dbGaP Study Accession: phs000178) available in a public repository the Genomic Data Commons Data Portal at https://portal.gdc.cancer.gov/. All the other data supporting the findings of this study are available within the article, the Supplementary Data1 files, the Supplementary Data 2, Supplementary Figures for uncropped blots and from the corresponding author upon reasonable request. The raw data and R-code files employed for generation of the Figure 1 and Suppl. Figure 1 data are deposited in Supplementary Data 1, the R-code is available at https://github.com/Bakin-lab/BRCA-BER.

Field-specific reporting

Please select the one below that is the best fit for your research. If you are not sure, read the appropriate sections before making your selection.

☒ Life sciences ☐ Behavioural & social sciences ☐ Ecological, evolutionary & environmental sciences

For a reference copy of the document with all sections, see nature.com/documents/nr-reporting-summary-flat.pdf

Life sciences study design

All studies must disclose on these points even when the disclosure is negative.

Sample size	For animal studies: A conservative sample size calculation is based on comparing the mean tumor size at a given time-point between treatment and control using a one-sided t-test. If we assume the control group has a mean tumor size of 1000mm3 and a standard deviation of 260mm3 (based on preliminary data), then n=8 mice/group has 85% power (at α=0.05) to detect this minimum reduction. Time to tumor-progression is analyzed using Kaplan-Meier methods, comparisons made by the log-rank test.
Data exclusions	No data were excluded from the analysis.
Replication	Experiments were repeated at least 2 times; animal studies included 8 mice per group.
Randomization	In animal studies, tumor-bearing animals were randomly assigned to treatment groups.
Blinding	Blinding in western blot data collection: investigators were blinded for data assignment to groups as sample loading settings were done prior to loading of gel, and immunoblot development.<br>Flow cytometry experiments were done by two independent investigators.

Reporting for specific materials, systems and methods

We require information from authors about some types of materials, experimental systems and methods used in many studies. Here, indicate whether each material, system or method listed is relevant to your study. If you are not sure if a list item applies to your research, read the appropriate section before selecting a response.

Materials & experimental systems

n/a	Involved in the study	
☐	☒	Antibodies
☐	☒	Eukaryotic cell lines
☒	☐	Palaeontology and archaeology
☐	☒	Animals and other organisms
☒	☐	Human research participants
☒	☐	Clinical data
☒	☐	Dual use research of concern

Methods

n/a	Involved in the study	
☒	☐	ChIP-seq
☐	☒	Flow cytometry
☒	☐	MRI-based neuroimaging

Antibodies

Antibodies used

anti-GAPDH (rabbit, polyclonal) Santa Cruz Biotechnology Cat# sc-25778; RRID: AB_10167668
anti-p53 (Mouse Monoclonal antibody, Clone do-1) Santa Cruz Biotechnology Cat# sc-126; RRID:AB_628082
anti-p21 (CDKN1A) (C-19, rabbit polyclonal) Santa Cruz Biotechnology Cat# sc-397; RRID:AB_632126
anti-RAD51 (H-92, rabbit polyclonal) Santa Cruz Biotechnology Cat# sc-8349; RRID:AB_22253533
anti-phospho-Ser15 p53 (rabbit, polyclonal) Cell Signaling Technology Cat# 9284; RRID:AB_331464
anti-PAR (mouse monoclonal anti-PADPr, Clone 10h) Santa Cruz Biotechnology Cat# sc-56198, RRID:AB_785249
anti-PARP1 (Mouse monoclonal antibody, Clone 5a5) Santa Cruz Biotechnology Cat# sc-56197; RRID:AB_630080
anti-phospho-Ser139 H2AX (γH2AX; rabbit polyclonal) Abcam Cat# ab-11174; RRID:AB_297813
anti-phospho-Ser139 H2AX (γH2AX; mouse mAb, JBW301) Millipore Cat# 05-636; RRID:AB_309864
anti-UNG Abcam Cat# ab23926; RRID:AB_778737
anti-MDM2 Santa Cruz Biotechnology Cat# sc-965; RRID:AB_627920
Goat Anti-Mouse IgG (H L)-HRP Conjugate antibody Bio-Rad Cat# 170-6516; RRID:AB_11125547
Goat Anti-Rabbit IgG (H L)-HRP Conjugate antibody Bio-Rad Cat# 170-6515; RRID:AB_11125142
anti-BrdU (Bu20a) Dako Cat# M0744; RRID:AB_10013660
Cleaved Caspase-3 (Asp175) Antibody Cell Signaling Technology Cat# 9661; RRID:AB_2341188
Rabbit monoclonal anti-Ki67 (clone SP6) ThermoFisher Cat#RM-9106-S1, RRID:AB_149792
anti-alpha-Tubulin mouse mAb, Clone B-5-1-2 Sigma-Aldrich Cat# T6074, RRID:AB_477582
Biotinylated secondary anti-rat antibody (goat) BD Biosciences Cat#559286, RRID:AB_397214

Validation	All antibodies were validated with appropriate references at the manufacturer's website.

Eukaryotic cell lines

Policy information about cell lines

Cell line source(s)	ATCC: MCF10A, MDA-MB-231, MDA-MB-468, BT549, CAL51, A549, WI-38, EMT6, 4T1; MCF10A-p21ko cell lines were from Dr. Ben Ho Park (Vanderbilt University)
Authentication	All cell lines were authenticated using short tandem repeat profiling by ATCC or the Roswell Park Core within the last three years.
Mycoplasma contamination	All studies were made using mycoplasma-free cells.
Commonly misidentified lines (See ICLAC register)	none

Animals and other organisms

Policy information about studies involving animals; ARRIVE guidelines recommended for reporting animal research

Laboratory animals	Female SCID/CB17 mice (6-7-week-old)
Wild animals	None
Field-collected samples	None
Ethics oversight	A study protocol and guidelines approved by the Institute Animal Care and Use Committee (IACUC). The facility is certified by the American Association for Accreditation of Laboratory Animal Care (AAALAC) and is in accordance with current regulation and standards of the US Department of Agriculture and the US Department of Health and Human Services.

Note that full information on the approval of the study protocol must also be provided in the manuscript.

Flow Cytometry

Plots

Confirm that:

- [x] The axis labels state the marker and fluorochrome used (e.g. CD4-FITC).
- [x] The axis scales are clearly visible. Include numbers along axes only for bottom left plot of group (a 'group' is an analysis of identical markers).
- [x] All plots are contour plots with outliers or pseudocolor plots.
- [x] A numerical value for number of cells or percentage (with statistics) is provided.

Methodology

Sample preparation	For cell cycle analysis, 300,000 cells were collected and fixed for 2 hrs in ice-cold 70% ethanol and stained for 2 hrs at 4°C in Krishan DNA Buffer (propidium iodide, sodium citrate, RNase A, NP40, and 0.1 mM HCl).
Instrument	BD LSRFortessa
Software	data are collected by FACSDiva (Version 6.1.3), and analyzed using ModFit Lt software (Version 5.0.9).
Cell population abundance	over 95% of cells were subjected to the analysis
Gating strategy	single cells were used in the analysis, and cell doublets were excluded

- [x] Tick this box to confirm that a figure exemplifying the gating strategy is provided in the Supplementary Information.
